# Sentiment Analysis on Online Videos by Time-Sync Comments

**DOI:** 10.3390/e25071016

**Published:** 2023-07-02

**Authors:** Jiangfeng Li, Ziyu Li, Xiaofeng Ma, Qinpei Zhao, Chenxi Zhang, Gang Yu

**Affiliations:** 1 School of Software Engineering, Tongji University, Shanghai 201804, China; lijf@tongji.edu.cn (J.L.); 2031540@tongji.edu.cn (Z.L.); qinpeizhao@tongji.edu.cn (Q.Z.); xzhang2000@163.com (C.Z.); 2 School of Electronic and Information Engineering, Tongji University, Shanghai 201804, China; 3 SILC Business School, Shanghai University, Shanghai 201800, China; gyu@shu.edu.cn; 4 SHU-SUCG Research Centre for Building Industrialization, Shanghai University, Shanghai 200072, China

**Keywords:** sentiment analysis, time-sync comments, video highlight extraction, sentimental intensity calculation

## Abstract

Video highlights are welcomed by audiences, and are composed of interesting or meaningful shots, such as funny shots. However, video shots of highlights are currently edited manually by video editors, which is inconvenient and consumes an enormous amount of time. A way to help video editors locate video highlights more efficiently is essential. Since interesting or meaningful highlights in videos usually imply strong sentiments, a sentiment analysis model is proposed to automatically recognize sentiments of video highlights by time-sync comments. As the comments are synchronized with video playback time, the model detects sentiment information in time series of user comments. Moreover, in the model, a sentimental intensity calculation method is designed to compute sentiments of shots quantitatively. The experiments show that our approach improves the F1 score by 12.8% and overlapped number by 8.0% compared with the best existing method in extracting sentiments of highlights and obtaining sentimental intensities, which provides assistance for video editors in editing video highlights efficiently.

## 1. Introduction

With the boom of online video websites, more and more people are likely to watch videos online. Those websites not only bring convenience in watching videos but also provide functions for people to make comments on videos. However, since a huge amount of videos are uploaded to the websites every day, it is hard for one to watch every minute in the videos. In this circumstance, audiences may prefer to watch video highlights, which are composed of excellent video fragments instead of watching entire videos.

Video highlights are a crucial aspect of video content as they provide audiences with a condensed version of the most interesting and meaningful parts of the video. However, the process of manually editing these highlights is time-consuming and labor-intensive, making it essential to find a more efficient way to locate the video highlights. In recent years, sentiment analysis has emerged as a promising approach of automatically recognizing sentiments of video highlights using time-sync comments.

Time-sync comments (TSCs) are messages that users send while watching a video to express their thoughts and feelings about what they are seeing. These comments appear on the screen at the moment they are made and reflect the users’ mood during that particular segment of the video. By analyzing the time-sync comments, we can gain insights into the emotions of the viewers and even predict the emotional trajectory of the video. In this paper, we mainly conduct experiments on Chinese time-sync comments. These comments are often used to express various emotions and moods, ranging from happiness and excitement to sadness and frustration. For example, viewers may leave comments like “OMG” or “lol” to express their amusement or laughter, while comments such as “so sad” or “heartbreaking” can indicate a feeling of sadness or sympathy.

By analyzing the sentiment of time-sync comments, we can detect sentiment information in the time series of comments and use this information to extract the most interesting or meaningful parts of the video. Furthermore, we can quantify the sentimental intensity of these shots using a sentimental intensity calculation method.

In this paper, we propose a TSC-based sentiment analysis model to extract highlights from videos and calculate their sentiment intensity. The main contributions include: (1) a sentiment fragments detection model for videos using TSC data is proposed to detect video fragments with strong sentiment from videos, (2) a highlight extraction strategy is designed to find video highlights, and (3) a sentiment intensity calculation method for video fragments is constructed in order to compute sentiments of video fragments quantitatively.

The rest of the paper is organized as follows. Section 2 reviews the related work. Section 3 defines two problems of sentiment analysis on online videos. Two sentiment analysis strategies using TSC are proposed in Section 4 and Section 5. Section 6 evaluates the performance of the model using a TSC dataset. We conclude our work in Section 7.

## 2. Related Work

### 2.1. Time-Sync Comments

Time-sync comments, first introduced in academia [1], are widely used in video websites, such as Acfun, Bilibili, and YouKu, which are some of the most popular video websites in China. One TSC is composed of a comment and a time stamp. It is a comment by an audience, which shows the audience’s opinion on a video shot.

The time stamp is synchronized to the shot’s playback time in the video [2]. TSCs are used for video classification tasks [3]. Current researchers use TSCs to extract video highlights [4,5,6]. Moreover, current approaches are beginning to apply TSCs to the emotional analysis of videos [7,8]. Bonifazi et al. [9] take into account the similarity between patterns and put forth a content semantic network called CS-Net to handle reviews. To measure the similarity between two networks, they calculated the similarity of structural features across different networks. As TSCs of a video indicate opinions of audiences on the shots of the video, text analysis of the TSCs is able to extract details for every single shot of a video. Moreover, the extraction results reflect not only explicit information but also implicit information.

### 2.2. Video Highlight Extraction

The work of video highlight extraction is mainly carried out by editors of online video websites manually. In order to extract highlights in videos, those editors have to watch the whole videos first. Then, they select video fragments that are interesting and may be welcomed by audiences. Lastly, the video fragments are re-edited and re-organized as video highlights. As such work is inefficient, it is necessary to provide a method that can extract interesting video fragments automatically. Recently, some researchers have begun to use TSCs for video highlight extraction. One work proposes to use “global + local” sentiment analysis to find highlights [5]. Another work proposes to use lag-calibration and the combination of topic and emotion concentration in an unsupervised way to detect highlights [6]. Actually, in a video, fragments that are welcomed by audiences always indicate one or more sentiments strongly. Therefore, in achieving the goal of welcomed video fragment extraction, sentiment detection for video fragments is the key process.

### 2.3. Sentiment Analysis

Many researchers have focused on detecting sentiment using image-based approaches. A number of researchers track the human face [10,11,12,13] or human pose [14,15,16,17,18], while some other researchers extract semantic features of sentiment from images [19,20,21,22,23,24]. However, compared with text-based processes, image-based approaches consume more time and cost more computational resources, but achieve less accuracy [25]. Additionally, labels extracted by the image-based approaches can only reflect explicit sentiments [26]. By contrast, both explicit and implicit sentiments can be detected by analyzing audience comments using the text-based approaches.

As the textual approaches have those advantages, many efforts have been directed to text-based analysis [27,28,29,30,31,32,33,34,35,36]. Nevertheless, current approaches either assign sentiment tags to whole videos instead of a single shot [37] or treat the video shots as independent objects [38], while a video segment constitutes a group of the shots that may have relations with preceding and following shots. Bonifazi et al. [39] propose a general framework capable of analyzing the range of sentiment associated with any topic on any social network.

In conclusion, while researchers primarily focus on tasks such as video classification and video clip recommendation using TSCs, they often overlook the potential of using TSCs for video highlight extraction and calculating the sentimental intensity of those highlights. Therefore, we propose a four-step strategy for extracting sentiment highlights in videos, which involves identifying and grouping together adjacent video fragments that share similar sentiment. Moreover, we introduce a strategy for quantitatively measuring the sentimental intensity of a highlight, taking into account not only the types of sentiment implied but also the strength of the sentiment within each type. By employing these strategies, we aim to enhance the understanding and representation of contents having various sentiment within videos.

## 3. Problem Definition

### 3.1. Illustration of Time-Sync Comments

A time-sync comment is composed of text-based comments and time stamps. The comment is usually a sentence of fewer than 20 words. Sometimes it is a text symbol representing an emotion, such as *OMG* standing for surprise, *LOL* meaning happiness, and *233333* expressing a laugh in habits of people who are using TSCs. The time stamp records the playback time of a video shot, and it is synchronized to the comments on the shot.

**Figure 1:**
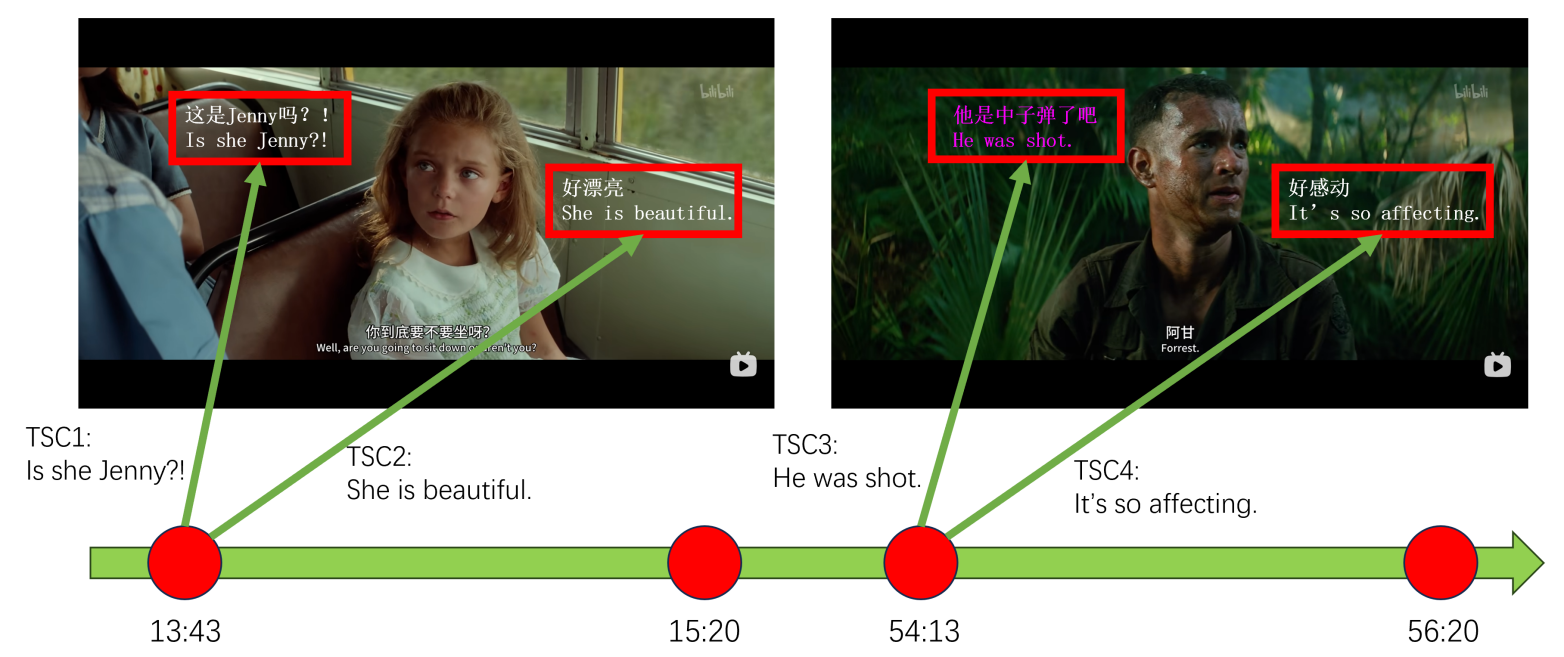
An Example of Time-Sync Comments in the Video Forrest Gump.

Figure 1 shows an example of two shots and their TSCs in the video *Forrest Gump*. In the figure, *Is she Jenny?!* and *She is beautiful* are two TSCs on the shot whose playback time is *13:43*, and *He was shot* and *It’s so affecting* are another two TSCs that are synchronized to the time stamp *54:13*.

The sentiment features of a video shot are indicated by TSCs. For example, *She is beautiful* reflects that the sentiment of the current shot is close to LIKE rather than HATE. In addition, *It’s so affecting* means that the fragment close to the playback time *54:13* contains a positive sentiment instead of a negative one.

### 3.2. Formal Definition

Let *v* be a video. Let $T_{start}$ and $T_{end}$ be the start time and finish time of *v*, respectively. Let $T_{v}$ be the length of *v*. We have $T_{v}=T_{end}-T_{start}$.

Let $F_{v}=\left\{f_{v,1},f_{v,2},\ldots ,f_{v,N_{F}}\right\}$ be a set of fragments in *v*, where $f_{v,i}\left(1\leq i\leq N_{F}\right)$ is the *i*-th fragment and $N_{F}$ is the number of fragments. We use $T_{start,i}$ and $T_{end,i}$ to represent the start time and finish time of $f_{v,i}$. We define that, for any $f_{v,i}\in F_{v}$, the length of $f_{v,i}$ is $T_{f}=T_{end,i}-T_{start,i}$. For any $f_{v,i},f_{v,i+1}\in F_{v}$, there is a interval $I\;(I<T_{f})$ between the start time of $f_{v,i}$ and that of $f_{v,i+1}$. That is $I=T_{start,i+1}-T_{start,i}$. It means every two adjacent fragments have an $\left(T_{f}-I\right)$-length overlap. Thus, $T_{v}=I\times \left(N_{F}-1\right)+T_{f}$. Obviously, $N_{F}=\left\lceil \frac{T_{v}-T_{f}+I}{I}\right\rceil$. Usually, $T_{f}$ is far less than $T_{v}$, and *I* is less than $T_{f}$. Therefore, the number of fragments in *v* is approximately $\left\lceil \frac{T_{v}}{I}\right\rceil$.

Suppose $T_{f}$ is small enough that makes one fragment unable to display a complete highlight. It means that a fragment is only a part of a highlight. In another words, a highlight consists of more than one continuous fragment when $T_{f}$ is small.

Let $H_{v}=\left\{h_{v,1},h_{v,2},\cdots ,h_{v,N_{H}}\right\}$ be a set of highlights in *v*, where $h_{v,i}\left(1\leq i\leq N_{H}\right)$ is the *i*-th highlight and $N_{H}$ is the number of highlights. For any $h_{v,i}\in H_{v}$, $h_{v,i}={⋃}_{j=s}^{t}\left\{f_{v,j}\right\}$, where $f_{v,j}\left(s\leq j\leq t\right)$ is the *j*-th fragment in *v*.

Suppose there are *k* types of sentiments. Let $S=\{s_{1},s_{2},\cdots ,s_{k}\}$ be the set of sentiments. Sentiment intensity of a highlight, $h_{v,i}$, is defined as $E_{d,hv,i}=\left(e_{1},e_{2},\cdots ,e_{k}\right)$. It is a vector that shows intensity distribution in the *k* types of sentiments for the highlight $h_{v,i}$. For any $e_{j}\left(1\leq j\leq k\right)$, it is an intensity value of sentiment type $s_{j}$ in $h_{v,i}$.

Let $B_{v}$ be the set of TSCs in *v*, and $B_{fv,i}$ be the set of TSCs in $f_{v,i}$. For any TSC $b\in B_{v}$, *b* is described as a tuple $\left(w_{b},t_{b},u_{b}\right)$, where $w_{b}$ is *b*’s comment, which is a set of words or text symbols, $t_{b}$ is *b*’s time stamp, and $u_{b}$ represents a user ID of an audience who sends *b*. Let $N_{U}$ be the total number of audiences who send comments to *v*. Let $T_{sync}\left(w\right)$ be a time stamp that is synchronized to a comment *w*, and user(w) be the user who sends *w*. In the case of tuple $\left(w_{b},t_{b},u_{b}\right)$, $T_{sync}\left(w_{b}\right)=t_{b}$, $user\left(w_{b}\right)=u_{b}$.

The notations defined are listed in Table 1.

### 3.3. Problem Statement

Under the formal description, the problems of sentiment highlight extraction and sentiment intensity calculation are defined. The two problems are described as follows.

(1) Problem of Sentiment Highlight Extraction:
  Given *v* and $B_{v}$. For any $1<i<N_{F}$, to find $l_{i}$ and $r_{i}$ to satisfy all the constraint conditions below,
  a. $1\leq l_{i}<i$ and $1<r_{i}\leq N_{F}-i$;
  b. For any $i-l_{i}\leq k\leq i+r_{i}-1$, $f_{v,k}$ and $f_{v,k+1}$ have similar sentiment;
  c. $f_{v,i-l_{i}-1}$ and $f_{v,i-l_{i}}$ do not have similar sentiment;
  d. $f_{v,i+r_{i}}$ and $f_{v,i+r_{i}+1}$ do not have similar sentiment.
(2) Problem of Sentiment Intensity calculation:
  Given $H_{v}$, $B_{v}$, and *S*. For any $1\leq i\leq N_{H}$, find a vector $\left(e_{1},e_{2},\cdots ,e_{k}\right)$ that shows intensity distribution in $\left(s_{1},s_{2},\cdots ,s_{k}\right)$ for $h_{v,i}$, where $e_{j}\left(1\leq j\leq k\right)$ is the value of intensity in $s_{j}$ and $s_{j}\in S$.

As fragments in the same highlight reflect similar sentiment, the problem of highlight extraction is how to gather fragments that have similar sentiment together. If the problem is solved, we can obtain a set of highlights, $H_{v}={\left\{{⋃}_{j=i-l_{i}}^{i+r_{i}}\left\{f_{v,j}\right\}\right\}}_{\left\{1\leq i\leq N_{F}\right\}}$. It means $H_{v}$ is a set of elements that are ${⋃}_{j=i-l_{i}}^{i+r_{i}}\left\{f_{v,j}\right\}$ for every $i\;(1\leq i\leq N_{F})$. After removing redundancy, $H_{v}$ can be organized in the format of $\left\{h_{v,1},h_{v,2},\ldots ,h_{v,N_{H}}\right\}$. After obtaining the set of highlights, $H_{v}$, the sentiment intensity of each highlight in $H_{v}$ can be computed by solving the problem of highlight sentiment intensity calculation using the TSC set.

## 4. Sentiment Highlight Extraction

**Figure 2:**
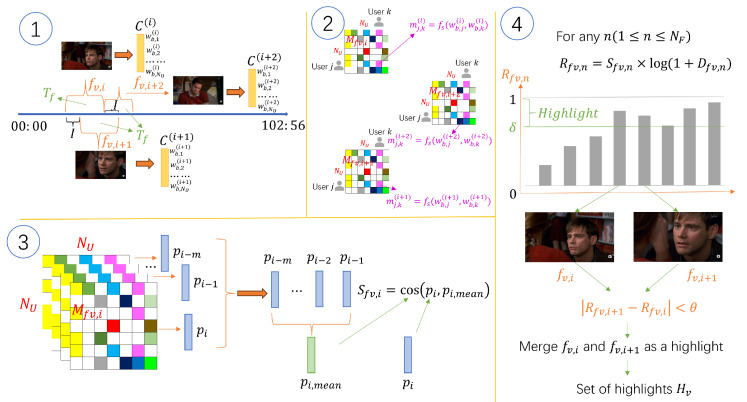
Processes of sentimental highlight extraction.

A strategy of sentiment highlight extraction is used to extract highlights in a video by gathering video adjacent fragments that have similar sentiment together. It is mainly composed of four steps: (1) TSC vectors of all fragments are constructed, (2) similarity matrices of all fragments are generated to measure similarities among user comments, (3) feature similarity of each fragment is calculated, and (4) the highlight score of each fragment is calculated. The processes of the strategy are shown in Figure 2. The details of the four steps in Figure 2 are described in the four subsections in Section 4.

### 4.1. Construct TSC Vectors

We construct a TSC vector, $C^{\left(i\right)}$, for fragment $f_{v,i}\left(1\leq i\leq N_{F}\right)$. It is organized as $C^{\left(i\right)}=\left(w_{b,1}^{\left(i\right)},w_{b,2}^{\left(i\right)},\ldots ,w_{b,N_{U}}^{\left(i\right)}\right)$. Each element, $w_{b,j}^{\left(i\right)}\left(1\leq j\leq N_{U}\right)$, is a set of comments on $f_{v,i}$, commented by user $u_{b_{j}}$. We describe $w_{b,j}^{\left(i\right)}$ as $w_{b,j}^{\left(i\right)}=\left\{w_{b}|T_{start,i}\leq T_{sync}\left(w_{b}\right)\leq T_{end,i},user\left(w_{b}\right)=u_{b_{j}}\right\}$, where $T_{start,i}$ and $T_{end,i}$ are the start time and finish time of fragment $f_{v,i}$, respectively.

### 4.2. Generate Similarity Matrices

A similarity matrix is generated for each fragment. It reflects the similarities of comments from different users on the same fragment. A similarity matrix, $M_{fv,i}$, has a size of $N_{U}\times N_{U}$. Let $m_{j,k}^{\left(i\right)}$ be the elements at the *j*-th row and *k*-th column in $M_{fv,i}$. Then, $m_{j,k}^{\left(i\right)}$ is calculated by the formula $m_{j,k}^{\left(i\right)}=f_{s}\left(w_{b,j}^{\left(i\right)},w_{b,k}^{\left(i\right)}\right)$, where $f_{s}\left(\cdot ,\cdot \right)$ is a similarity factor such as cosine similarity, and $w_{b,j}^{\left(i\right)}$ and $w_{b,k}^{\left(i\right)}$ are two sets of comments on $f_{v,i}$ commented by user $u_{b_{j}}$ and user $u_{b_{k}}$, respectively.

### 4.3. Calculate Feature Similarity

After obtaining the similarity matrix $M_{fv,i}$ for fragment $f_{v,i}$, we easily obtain $M_{fv,i}$’s largest real eigenvalue and its corresponding eigenvector, $p_{i}$. The Perron–Frobenius theorem ensures that components in $p_{i}$ are positive values. Values in $p_{i}$ are thought of as features of “sentiment” implied by audiences’ comments on fragment $f_{v,i}$.

Since $p_{i}$ represents features of $f_{v,i}$, we calculate the mean value of features of the nearest *m* fragments before $f_{v,i}$. The mean value, $p_{i,mean}$, is calculated by Equation (1).

(1) $$p_{i,mean}=\left\{\begin{array}{cc} \left(\sum_{j=1}^{i-1}p_{j}\right)/\left(i-1\right) & i\leq m\\ (\sum_{j=i-m}^{i-1}p_{j})/m & i>m \end{array}\right.$$

The feature similarity of fragment $f_{v,i}$, notated as $S_{fv,i}$, is the similarity of $p_{i}$ and $p_{i,mean}$. The similarity is calculated using the cosine function, which is $S_{fv,i}=\cos\left(p_{i},p_{i,mean}\right)$.

### 4.4. Finding Video Highlights

Firstly, highlight scores of all fragments are calculated in order to decide which fragments are put together in the same highlight. $R_{fv,n}$, the highlight score of fragment $f_{v,n}$, is calculated by Equation (2), where $D_{fv,n}$ is the TSC density in $f_{v,n}$, defined as the number of all TSCs commented on, $f_{v,n}$.

(2) $$R_{fv,n}=S_{fv,n}\times \log\left(1+D_{fv,n}\right)$$

The larger TSC density a fragment has, the stronger sentiment the fragment manifests. It is attributed to the fact that people prefer to express their opinions when they feel a fragment is interesting or meaningful, which makes the number of TSCs increase.

Next, fragments that have high highlight scores are selected as single highlights.

A highlight score of a fragment indicates the possibility that the fragment is considered as a highlight. The higher a fragment’s highlight score is, the higher is probability that the fragment may become a highlight.

A highlight threshold, δ, is set for single highlight detection. If $R_{fv,i}$, the highlight score of fragment $f_{v,i}$, is larger than the highlight threshold, δ, and $f_{v,i}$ is selected as a single highlight.

After that, relevant single highlights are merged into one highlight. For any two fragments, $f_{v,i}$ and $f_{v,j}$, they will be merged as a highlight if (a) $R_{fv,i}>\delta$, (b) $R_{fv,j}>\delta$, (c) |i−j|=1, and (d) $|R_{fv,i}-R_{fv,j}|<\theta$, where δ is the highlight threshold, and θ is a link threshold for deciding whether two fragments have strong relevance in sentiment.

Under the strategy, a fragment will be merged with its neighboring fragment if the two fragments are relevant in sentiment and both of them are single highlights. Moreover, three or more adjacent fragments can be merged as a highlight.

Lastly, a highlight set is obtained by putting all the highlights together. We can obtain a highlight set, $H_{v}$, that composed of different highlights in video *v*. A highlight, $h_{v,i}$, in $H_{v}$ is called a sentiment highlight of $H_{v}$.

## 5. Sentiment Intensity Calculation

A strategy of sentimental intensity is used to measure the strength of sentiment for a highlight quantitatively. It reflects not only which sentiment types the highlight implies, but also how strong the highlight’s sentiment is in each type. In this paper, we choose TSCs in Chinese language to analyze sentiment intensity because Chinese is the most popular language in TSCs. For TSCs in other languages, the sentiment intensity can still be calculated using grammar rules of the languages and conventional sentiment analysis methods such as Bidirectional Encoder Representations from Transformers (BERT) in the same way.

### 5.1. Word Groups Division for TSCs

Using the strategy of sentiment highlight extraction, a set of highlights, $H_{v}$, is extracted from video *v*. For a highlight $h_{v,i}\in H_{v}$, it is composed of one or more adjacent fragments. That is, $h_{v,i}={⋃}_{j=s_{i}}^{s_{i}+N_{i}-1}\left\{f_{v,j}\right\}$, where $N_{i}$ is the number of fragments in $h_{v,i}$, $s_{i}$ is the index of the first fragment in $h_{v,i}$, and $s_{i}+N_{i}-1$ is the index of the last fragment in $h_{v,i}$.

Let $CMT_{hv,i}$ be a set of TSC comments that are commented in fragments of $h_{v,i}$. Thus, $CMT_{hv,i}=\left\{w_{b}|T_{start,s_{i}}\leq T_{sync}\left(w_{b}\right)\leq T_{end,s_{i}+N_{i}-1}\right\}$, where $T_{start,s_{i}}$ and $T_{end,s_{i}+N_{i}-1}$ are the start time of $f_{v,s_{i}}$ and the finish time of $f_{v,s_{i}+N_{i}-1}$, respectively.

Through linguistic analysis, sentiments implied in a sentence are impacted by some special words in the sentence. In the case of TSCs, there are three categories of special words, which are emotional words, adverbs, and negative words. An emotional word in comments expresses some kinds of sentiments and their intensity. An adverb strengthens or weakens sentiment intensity for a comment. A negative word changes the meaning of a comment completely. For example, both the sentences *I am a little bit happy* and *I am very happy* express the sentiment of HAPPY, but the sentiment of the second sentence is much stronger than that of the first one. It is attributed to the fact that *very* is an adverb whose weight is much greater than *a little bit*. Another example, *I am happy,* shows the sentiment of HAPPY, while *I am not happy* describes a sentiment opposite to HAPPY, i.e., probably SAD.

Emotional words in $CMT_{hv,i}$ can be selected according to a dictionary of emotional words. Sentiment intensities of the emotional words can also be obtained from the dictionary. Actually, for an emotional word, $d_{j}$, its sentiment intensity, $E_{d,dj}=\left(e_{1},e_{2},\ldots ,e_{k}\right)$, is a distribution of sentiment strengths on the *k* types of sentiments, and $e_{j}\left(1\leq e_{j}\leq k\right)$ is the strength of $d_{j}$ on the *j*-th sentiment type.

Most words in TSCs can be covered by the dictionary. However, there are some new terms that are not included in the dictionary. For those emotional terms that exist in $CMT_{hv,i}$ but are not found in the dictionary, we extend the dictionary by setting a sentiment type and a value of sentiment intensity. There are two available approaches to extend the sentiment dictionary. One method uses a dictionary of synonyms, and new terms are synonymous with existing ones. We replace new terms with terms from the existing sentiment dictionary, thus obtaining a similar sentiment intensity. Another approach uses the original sentiment dictionary as a foundation and calculates the semantic similarity between new terms and those terms in the sentiment dictionary. It allows for the extension of the sentiment dictionary based on the semantic associations between terms. As the dictionary extension approaches are beyond the topics of this paper, it will not be introduced in the details of approaches in this paper.

Like a sentiment intensity, $E_{d,dj}$, of an emotional word, $d_{j}$, can be obtained from the dictionary of emotional words, a weight, $W_{D}$, for an adverb, *D*, can be obtained through a dictionary of adverbs. Similarly, negative words in $CMT_{hv,i}$ are able to be found easily from a dictionary of negative words.

**Figure 3:**
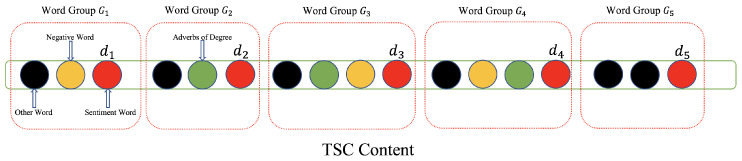
Groups of TSC words.

Suppose there are $N_{D,i}$ emotional words in $CMT_{hv,i}$, and the words of comments in $CMT_{hv,i}$ are organized into $N_{D,i}$ groups $\left\{G_{1},G_{2},\cdots ,G_{N_{D,i}}\right\}$. Each emotional word with its related adverbs and negative words are put into the same group. Thus, every group contains only one emotional word and may include one or more adverbs and negative words. Figure 3 shows groups of TSC words.

### 5.2. Sentiment Intensity Calculation for Highlights

According to the definition in Section 2, $E_{d,hv,i}=\left(e_{1},e_{2},\cdots ,e_{k}\right)$ is the sentiment intensity of highlight $h_{v,i}$, where *k* is the number of sentiment types, and $e_{j}\left(1\leq j\leq k\right)$ is an intensity value of the *j*-th sentiment type in $h_{v,i}$.

The sentiment intensity of $G_{j}\left(1\leq j\leq N_{D,i}\right)$ is affected by adverbs and negative words in $G_{j}$. The sentiment intensity of $G_{j}$ is calculated in three situations:

(a) There is neither an adverb nor negative word in $G_{j}$. The sentiment intensity of $G_{j}$ is the same as that of emotional word $d_{j}$, which is
  $$E_{d,Gj}=E_{d,dj}$$
  where $E_{d,dj}$ is the sentiment intensity of emotional word $d_{j}$.
(b) There is no adverb but there are $N_{n}\left(N_{n}\geq 1\right)$ negative words in $G_{j}$. Since a negative word oppositely affects a emotional word, in Chinese grammar, the presence of an even number of negative words at the same time indicates a stronger positive meaning, while the simultaneous appearance of an odd number of negative words indicates a stronger negative meaning. Therefore, according to the number of negative words that appear, the sentiment intensity of $G_{j}$ is calculated as
  $$E_{d,Gj}={\left(-1\right)}^{N_{n}}\times E_{d,dj}$$
  where $E_{d,dj}$ is the sentiment intensity of emotional word $d_{j}$.
(c) There is no negative word but there is one adverb in $G_{j}$. The sentiment intensity of $G_{j}$ is calculated as
  $$E_{d,Gj}=W_{D}\times E_{d,dj}$$
  where $W_{D}$ is the weight of adverb *D*, and $E_{d,dj}$ is the sentiment intensity of emotional word $d_{j}$.
(d) There are both adverbs and $N_{n}\left(N_{n}\geq 1\right)$ negative words in $G_{j}$. As comments in $CMT_{hv,i}$ are Chinese characters, according to Chinese linguistic features, if there is more than one adverb in word group $G_{j}$, then we consider $G_{j}$ to be not grammatical, so we just consider that there is one adverb or less in $G_{j}$. At the same time, an adverb written before or after a negative word affects the sentiment intensity of a word group differently. If the position of an adverb is before all negative words in $G_{j}$, the sentiment intensity of $G_{j}$ is calculated as
  $$E_{d,Gj}={\left(-1\right)}^{N_{n}}\times W_{D}\times E_{d,dj}$$
  where $W_{D}$ is the weight of adverb *D*, and $E_{d,dj}$ is the sentiment intensity of emotional word $d_{j}$.
  If there are $N_{n_{1}}\left(1<N_{n_{1}}\leq N_{n}\right)$ negative words before *D*, and $N_{n_{2}}\left(N_{n_{2}}=N_{n}-N_{n_{1}}\right)$ negative words after *D*, the sentiment intensity of $G_{j}$ is calculated as
  $$E_{d,Gj}={\left(-1\right)}^{N_{n_{1}}+1}\times W\times W_{D}\times {\left(-1\right)}^{N_{n_{2}}}\times E_{d,dj}$$
  where *W* is the parameter to weaken sentiment intensity, $W_{D}$ is the weight of adverb *D*, and $E_{d,dj}$ is the sentiment intensity of emotional word $d_{j}$.

From the processes above, we can obtain the sentiment intensity of each word group, $G_{j}$, in $CMT_{hv,i}$. Then, we use the sentiment intensity of all word groups to generate the sentiment intensity of a video highlight. The sentiment intensity of highlight, $h_{v,i}$, is calculated as

$$E_{d,hv,i}=\frac{\sum_{j=1}^{N_{D,i}}E_{d,Gj}}{(T_{end,s_{i}+N_{i}-1}-T_{start,s_{i}})/I}$$

where $E_{d,Gj}$ is the *j*-th word group in $CMT_{hv,i}$, $N_{D,i}$ is the number of word groups in $CMT_{hv,i}$, $T_{start,s_{i}}$ and $T_{end,s_{i}+N_{i}-1}$ are the start point and end point of video highlight $h_{v,i}$, respectively, and *I* is the interval between $T_{start,s_{i}}$ and $T_{start,s_{i+1}}$.

The sentiment intensity, $E_{d,hv,i}$, is an average value of total sentiment intensity in the highlight, $h_{v,i}$, per unit time.

## 6. Evaluation

### 6.1. Experiment Setup

A TSC dataset that includes approximate 16 million TSCs is used to evaluate the performance of our proposed work. The TSCs are collected from 4841 online videos, which contain movies, animation, TV series and variety shows.

Emotional words ontology (http://ir.dlut.edu.cn/info/1013/1142.htm (accessed on 1 May 2023)), provided by the Dalian University of Technology, is used to build up our sentiment dictionary. In the dictionary, each word is related to a sentiment intensity, a 7-dimension vector. Each dimension represents one of seven kinds of sentiment, which are *happy*, *good*, *angry*, *sad*, *afraid*, *hate*, and *shock*.

We randomly selected 34 movies on the Bilibili website, including action movies, comedy movies, fantasy movies, horror movies, etc. The TSCs of movies including *Spider-Man: Homecoming*, *Harry Potter and the Philosopher’s Stone*, *Green Book*, *Charlie Chaplin*, *The Shawshank Redemption*, *Secret Superstar*, etc. from the dataset were chosen for our experiments. In the experiments, fragment length $T_{f}$ is set to 30 s and fragment interval *I* is set to 20 s. Different movies have different numbers of time-sync comments. We randomly selected 5000 time-sync comments for each movie. We combined the movie categories on the iMDb website and the sentiment analysis of all the time-sync comments of the movies to classify the selected movies in the experiments. The basic information of the movies is shown in Table 2.

There are some highlights in each movie. All of the baseline highlights are manually selected by movie audiences. We obtained the edited highlight moment video on the imdb and bilibili websites, and matched it with the original movie to obtain the highlight time. The baseline highlights of some movies in the dataset are listed in Table 3. We chose one movie from each category, and we can find the movie name, highlight number, and highlight playback time in Table 3.

In the experiments, we used two metrics to measure the performance of sentiment highlight extraction strategy, which are,

(1) Sentiment highlight F1 score, calculated by equation
  $$F1\;Score=2\times \frac{precision\times recall}{precision+recall}$$
(2) Overlapped number count, which is the number of overlapped fragments between highlights extracted by our proposed approach and the baseline highlights.

### 6.2. Evaluation of Sentiment Highlights Extraction

In the experiments, the highlight threshold, δ∈[0,1), and linking threshold, θ∈[0,1), are two adjustable parameters. After a number of experiments, results show that θ has little effect on sentiment highlight extraction. Therefore, θ is set 0.1 in the experiments. In order to obtain the optimal value of δ, we calculated the average F1 score and overlapped number count under different δ. We used Latent Dirichelet Allocation (LDA) and BERT, respectively, to construct TSC vectors in our method. The main parameters in the LDA model are as follows: the number of theme sampling iteration η=100; and the quantity of hidden topics K=100. The main parameters in the BERT model are as follows: the number of hidden layers is 12; the hidden size is 768; and the number of attention heads is 12. We compared our method with three methods: (1) randomly selected fragments, (2) Multi-Topic Emotion Recognition (MTER) [5], and (3) the method proposed by Ping [6]. We also compared our methods using different ways for constructing TSC vectors and the method without the step in section find video highlights. The overlapped number count is the sum of overlapped number from these methods.

**Figure 4:**
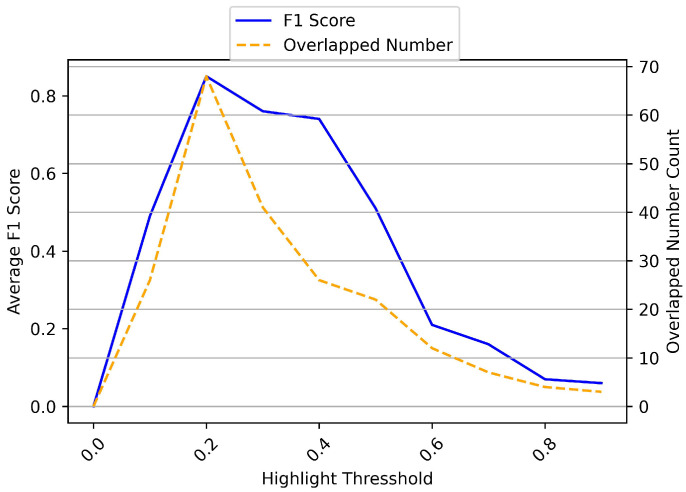
Average F1 score and overlapped number count.

Figure 4 shows the experiments results of the sentiment highlight extraction strategy. As we can see in the figure, our proposed strategy has the highest average F1 score and highest overlapped number count when δ=0.2; in the other words, our model has the optimal extraction effect at δ=0.2. Therefore, we set δ=0.2 and θ=0.1 in the following experiments.

Table 4 shows the sentiment highlight F1 score for these sentiment highlight extraction methods. The optimal value of each row is shown in bold. From the experimental results, it can be seen that, for different categories of movies, the experimental method has better experimental results with comedies and dramas, because the highlights of these movies are more concentrated, while, for action, horror and thriller movies, the experimental results are lower. On one hand, the sentiment type is relatively simple and single in comedy movies. Audiences have the same feeling when they watch happy clips. There is an agreement on the understandings of the clips. Therefore, the happy clips can be easily extracted as highlights, which makes the F1 score of the comedy genre higher than that of other genres. On the other hand, there are a greater number of various scenes in other genres, such as fighting in action movies, and jump scares in horror and thriller movies. The sentiment types are various and complex in those genres. It makes different audiences have different understandings, even when they watch the same scenes. Therefore, movies of those genres achieve a lower F1 score compared with comedy movies.

From the experimental results, we can see that our method with BERT has a higher F1 score than other methods with regards to action–adventure movies, comedies, fantasy movies, crime movies, and drama movies. This shows that our method has better universality for different categories of movies. However, in the genre of horror and thriller movies, the experimental results of our method are slightly worse than those proposed by Ping [6]. We speculate that this may be because people will be full of tension when watching horror and thriller movies, and there is a larger latency. Meanwhile, the values of the F1 score in our method are all greater than 0.5, while the method proposed by Ping performs poorly on some movies, such as *Slumdog Millionaire*. Therefore, our method performs more stably with respect to the method proposed by Ping. In summary, we find that the experimental results of our proposed method are better than MTER [5] and Ping [6]. The experimental results indicate that our method has a better performance with higher overall accuracy and F1 score than other methods. The experimental results show that our method has good results with different categories of movies, and has certain universality for various categories of movies.

**Figure 5:**
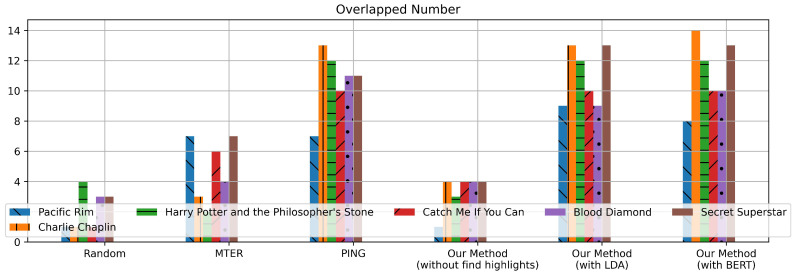
Sentiment highlight overlapped number.

As can be seen from Table 5, the average value of our method with the BERT overlapped number is better than other methods. We randomly selected one movie of each type from Table 2. The overlapped number of the movie is visually displayed in Figure 5. Figure 5 demonstrates that the overlapped number of our method with BERT is higher than other methods in most movies.

The results of our experiments show that different types of movies yield different results. Specifically, we found that the movie genre affects the emotional response of audiences, which in turn impacts the use of emotional words in the TSCs. For instance, the *Charlie Chaplin* and *Secret Superstar* overlapped numbers of these methods are high while the *Pacific Rim* overlapped numbers of these methods are low. *Charlie Chaplin* is a comedy movie with a relaxing and cheerful emotional tone, which increases the probability of audiences using straightforward emotional words such as “2333”, “funny”, and “interesting”. Similarly, in *Secret Superstar*, a movie with a profound conceptual theme, the plot twist can elicit a strong emotional response from audiences, leading them to express straightforward emotional words more frequently.

In contrast, for action movies such as *Pacific Rim*, audiences tend to pay more attention to fight scenes and special effects rather than the emotional content of the movie, resulting in a lower probability of using similar, straightforward emotional words. As a result, we observed a higher overlap in the emotional words used by audiences for *Charlie Chaplin* and *Secret Superstar*, and a lower overlap for *Pacific Rim*.

To investigate the influence of various similarity measures on experimental results, we conducted experiments using different similarity measures in conjunction with BERT. The employed measures encompassed the Euclidean distance, Pearson correlation coefficient, Manhattan distance, Minkowski distance, and cosine similarity. The experimental results, shown in Table 6, indicate that the employment of cosine similarity demonstrates a higher average F1 score and average overlapped number. Based on these results, we selected cosine similarity as the preferred measure for our method.

### 6.3. Evaluation of Sentiment Intensity Calculation

We randomly selected one movie of each type from Table 2 to show the experimental results of sentiment intensity. The experimental results of sentiment intensity information are listed in Table 7 after normalization (for each movie, three highlights are listed).

In Table 7, we can find the representative sentiment highlight information. We can see that, for different categories of movies, the distribution of sentiment on highlighted clips is not the same. In addition, these emotional distributions match our impressions of these movies. For instance, *Charlie Chaplin* is a comedy. The movie’s emotional fundamental key is relaxing, so the *good* dimension value is much higher than other dimensions. Furthermore, the sentiment intensity of *Secret Superstar* distributes on each dimension much more evenly instead of focusing on the same dimension. This is also in line with our expectations. *Secret Superstar* is a movie with various sentiments, which means its sentiment is complicated, and audiences may have quite different views on the same sentiment highlight.

To evaluate performance of the strategy of sentiment intensity calculation, we invited three experts who are professional in movie appreciation to label the sentiment intensity for each sentiment highlight.

After comparing sentiment highlights and intensities with their corresponding movie shots and origin TSC data, we found that our sentiment intensity can describe the sentiment information for sentiment highlights very well.

## 7. Conclusions and Future Work

In this paper, a time-sync-comments-based sentiment analysis model aimed at extracting sentiment highlights from videos and measuring sentiment intensity for highlights using TSCs is proposed. A four-step approach to extract video highlights and a strategy for calculating sentiment intensity are proposed, enabling the quantitative assessment of sentiment within these video highlights. The experimental results not only show that our approach improves the F1 score by 12.8% and overlapped number by 8.0% compared with the best existing method in highlight extraction, but also indicate a sentiment distribution in line with the corresponding movie scenes. Moreover, the proposed approach can be widely used for TSCs in various language. Strategies of sentiment highlight extraction and sentiment intensity calculation proposed in this paper focus on Chinese TSCs, but they can work on other languages by replacing grammar rules and sentiment analysis methods in other languages.

In the future, prior knowledge will be considered in highlight extraction strategy in order to improve the performances for those movie genres such as action, horror and thriller movies. Then, the sentiment dictionary will be continuously extended to increase the performances of sentiment intensity calculation.

## Figures and Tables

**Table 1:** Notation list.

Symbol	Description
*v*	TSC commented video
$T_{start}$	Start time of video *v*
$T_{end}$	Finish time of video *v*
$T_{v}$	Video length (time duration)
$F_{v}$	Set of fragments in video *v*
$N_{F}$	The number of fragments in video *v*
$f_{v,i}$	*i*-th fragment in video *v*
$T_{start,i}$	Start time of fragment $f_{v,i}$
$T_{end,i}$	Finish time of fragment $f_{v,i}$
$T_{f}$	Length of fragment (time span)
*I*	Interval between $T_{start,i}$ and $T_{start,i+1}$
$H_{v}$	Set of highlights in video *v*
$N_{H}$	The number of highlights in video *v*
$h_{v,i}$	*i*-th highlight in video *v*
*S*	Set of *k*-type sentiments
$s_{i}$	*i*-th type in sentiment set *S*
$E_{d,hv,i}$	Sentiment intensity of highlight $h_{v,i}$
$e_{i}$	Intensity value of sentiment type $s_{j}$
$B_{v}$	Set of TSCs in video *v*
$N_{B}$	The number of TSC in the video *v*
$B_{fv,i}$	Set of TSC in fragment $f_{v,i}$
*b*	One TSC in TSC set $B_{v}$
$w_{b}$	Comment of TSC *b*
$t_{b}$	Time stamp of TSC *b*
$u_{b}$	User who sends TSC *b*
$N_{U}$	The number of users who send TSCs in video *v*

**Table 2:** Basic information of movies.

Movie Name	Movie Length	Movie Type
Spider-Man: Homecoming	133 min 32 s	Action and Adventure
White Snake	98 min 42 s	Action and Adventure
Inception	148 min 8 s	Action and Adventure
Jurassic World Dominion	147 min 12 s	Action and Adventure
Pacific Rim	131 min 17 s	Action and Adventure
Transformers	143 min 23 s	Action and Adventure
Ready Player One	139 min 57 s	Action and Adventure
World War Z	123 min 3 s	Action and Adventure
Green Book	130 min 11 s	Comedy
Charlie Chaplin	144 min 30 s	Comedy
Let the Bullets Fly	126 min 38 s	Comedy
Johnny English	87 min 25 s	Comedy
Modern Times	86 min 43 s	Comedy
The Croods: A New Age	95 min 20 s	Comedy
La La Land	128 min 2 s	Comedy
The Truman Show	102 min 57 s	Comedy
Harry Potter and the Philosopher’s Stone	158 min 50 s	Fantasy
Fantastic Beasts and Where to Find Them	132 min 52 s	Fantasy
Kong	118 min 32 s	Fantasy
Triangle	98 min 59 s	Fantasy
The Shawshank Redemption	142 min 29 s	Crime
Catch Me If You Can	140 min 44 s	Crime
Slumdog Millionaire	120 min 38 s	Crime
Who Am I— Kein System ist sicher	101 min 47 s	Crime
Escape Room: Tournament of Champions	88 min 5 s	Horror
The Meg	114 min 38 s	Horror
Blood Diamond	143 min 21 s	Thriller
Shutter Island	138 min 4 s	Thriller
Secret Superstar	149 min 47 s	Music
Heidi	96 min 24 s	Family
Duo Guan	134 min 45 s	Sport
Saving Private Ryan	169 min 26 s	War
Source Code	93 min 18 s	Action
Dangal	139 min 57 s	Action

**Table 3:** Movies’ baseline highlights.

Movie Name	Highlight No.	Highlight Playback Time	Movie Name	Highlight No.	Highlight Playback Time
**Pacific** **Rim**	1	18:48–19:11	**Charlie** **Chaplin**	1	1:00–1:55
	2	20:20–21:00		2	4:07–4:34
	3	26:50–27:57		3	7:08–7:33
	4	52:09–52:56		4	9:01–9:39
	5	78:02–79:00		5	20:26–21:17
	6	79:26–80:51		6	23:41–24:10
	7	82:03–82:39		7	24:48–25:30
	8	89:01–90:12		8	30:06–30:54
	9	94:30–95:13		9	37:50–38:37
	10	96:02–96:53		10	39:05–39:58
	11	101:05–101:36		11	42:03–42:32
	12	113:49–114:15		12	45:23–45:57
	13	118:28–118:51		13	54:28–55:17
	14	121:09–122:40		14	55:48–56:39
				15	57:29–58:20
				16	94:49–95:33
				17	111:25–111:57
				18	118:01–118:50
				19	130:26–131:17
**Harry Potter** **and the** **Philosopher’s Stone**	1	0:00–1:30	**Catch Me** **If You Can**	1	1:25–1:54
	2	12:24–13:11		2	2:26–3:10
	3	13:45–14:58		3	20:29–20:55
	4	21:00–22:11		4	21:07–21:58
	5	23:47–24:11		5	24:06–24:35
	6	26:25–27:00		6	25:29–25:52
	7	36:46–37:40		7	26:20–26:54
	8	40:50–42:34		8	40:46–41:59
	9	48:27–48:57		9	55:45–56:10
	10	52:08–52:51		10	58:25–58:55
	11	53:21–53:59		11	59:50–60:20
	12	56:41–57:20		12	61:23–62:16
	13	66:01–66:39		13	75:49–76:12
	14	70:41–71:12		14	84:43–85:30
	15	77:41–78:32		15	107:48–108:17
	16	108:44–109:19		16	126:09–126:59
	17	147:23–148:18		17	127:28–128:20
	18	150:05–150:52		18	128:44–129:20
				19	134:24–135:50
**Blood** **Diamond**	1	6:40–7:20	**Secret** **Superstar**	1	53:45–54:35
	2	24:45–25:10		2	60:08–61:12
	3	49:23–49:59		3	66:41–67:12
	4	55:46–56:11		4	67:24–67:59
	5	60:23–61:30		5	72:21–72:53
	6	68:20–69:00		6	79:30–79:50
	7	72:10–73:16		7	81:26–81:51
	8	80:43–81:34		8	93:28–93:54
	9	91:27–92:19		9	96:00–97:10
	10	96:47–97:19		10	97:40–98:14
	11	108:04–108:57		11	102:41–103:35
	12	109:29–109:50		12	110:23–111:33
	13	110:45–111:10		13	132:05–132:50
	14	115:44–116:18		14	134:21–135:18
	15	128:10–129:12		15	138:30–139:12
	16	131:42–132:16		16	139:42–140:36
	17	132:47–133:11		17	144:28–144:59
	18	134:05–135:35		18	145:29–145:53
				19	146:01–146:32

**Table 4:** Sentiment highlight F1 score.

Movie Name	Random	MTER	PING	Our Method (without Find Highlights)	Our Method (with LDA)	Our Method (with BERT)
Spider-Man: Homecoming	0.100	0.200	0.597	0.167	0.364	**0.615**
White Snake	0.300	0.091	0.824	0.267	0.824	**0.828**
Inception	0.083	0.267	**0.650**	0.200	0.400	0.588
Jurassic World Dominion	0.133	0.062	0.520	0.356	0.571	**0.636**
Pacific Rim	0.071	0.467	0.579	0.110	0.707	**0.710**
Transformers	0.409	0.472	0.609	0.312	**0.733**	0.661
Ready Player One	0.214	0.366	**0.741**	0.268	0.600	0.606
World War Z	0.200	0.375	0.686	0.320	0.730	**0.733**
Green Book	0.200	0.091	**0.632**	0.267	0.571	0.591
Charlie Chaplin	0.126	0.150	0.742	0.253	0.813	**0.831**
Let the Bullets Fly	0.214	0.067	**0.649**	0.245	0.586	0.545
Johnny English	0.231	0.315	0.429	0.154	0.497	**0.770**
Modern Times	0.200	0.462	0.655	0.286	0.656	**0.750**
The Croods: A New Age	0.167	0.100	0.500	0.370	0.686	**0.717**
La La Land	0.250	0.154	0.642	0.111	0.737	**0.800**
The Truman Show	0.296	0.402	0.623	0.320	0.709	**0.714**
Harry Potter and the Philosopher’s Stone	0.105	0.121	0.699	0.150	0.733	**0.774**
Fantastic Beasts and Where to Find Them	0.190	0.211	0.606	0.074	**0.705**	0.638
Kong	0.389	0.392	0.759	0.303	0.800	**0.875**
Triangle	0.100	0.125	0.500	0.286	0.533	**0.625**
The Shawshank Redemption	0.167	0.286	**0.636**	0.222	0.500	0.515
Catch Me If You Can	0.158	0.271	0.525	0.268	**0.703**	0.606
Slumdog Millionaire	0.143	0.333	0.299	0.165	**0.707**	0.652
Who Am I - Kein System ist sicher	0.083	0.267	0.612	0.200	0.573	**0.575**
Escape Room: Tournament of Champions	0.100	0.091	**0.816**	0.267	0.750	0.773
The Meg	0.154	0.214	0.422	0.185	0.700	**0.742**
Blood Diamond	0.056	0.211	**0.747**	0.222	0.654	0.701
Shutter Island	0.171	0.267	**0.691**	0.390	0.600	0.610
Secret Superstar	0.158	0.375	0.620	0.180	**0.861**	0.796
Heidi	0.250	0.378	0.677	0.214	0.636	**0.653**
Duo Guan	0.133	0.343	0.456	0.170	0.549	**0.596**
Saving Private Ryan	0.247	0.211	0.693	0.267	0.759	**0.800**
Source Code	0.143	0.200	0.692	0.190	0.807	**0.923**
Dangal	0.176	0.167	0.472	0.299	0.626	**0.769**
Average	0.180	0.250	0.618	0.237	0.658	**0.697**

**Table 5:** Average overlapped number.

	Random	MTER	PING	Our Method (without Find Highlights)	Our Method (with LDA)	Our Method (with BERT)
Average overlapped number	2.23	4.10	7.71	2.91	8.20	**8.32**

**Table 6:** F1 score and overlapped number with different similarity measures.

	Euclidean Distance	Pearson Correlation Coefficient	Manhattan Distance	Minkowski Distance	Cosine Similarity
Average F1 Score	0.660	0.685	0.619	0.669	**0.697**
Average Overlapped Number	7.66	8.21	6.94	7.73	**8.32**

**Table 7:** Representative sentiment highlights.

	Pacific Rim			Charlie Chaplin		
Playback Time	26:50–27:57	96:02–96:53	121:09–122:40	37:50–38:37	45:23–45:57	94:49–95:33
Intensity Value	0.03,0.62,0.0,0.0, 0.02,0.34,0.0	0.07,0.24,0.0,0.12, 0.05,0.45,0.06	0.10,0.61,0.0,0.0, 0.09,0.20,0.0	0.05,0.93,0.0,0.0, 0.02,0.0,0.0	0.04,0.80,0.0,0.12, 0.0,0.04,0.0	0.08,0.63,0.0,0.11, 0.0,0.18,0.0
Intensity Figure	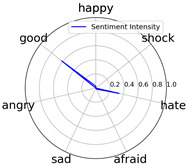	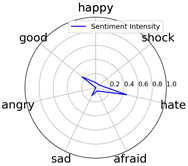	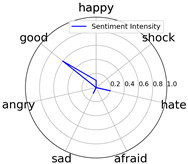	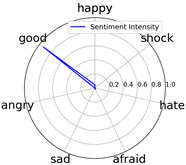	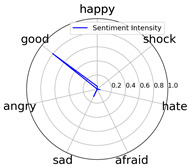	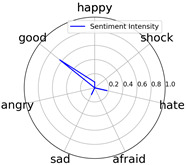
Film Plot	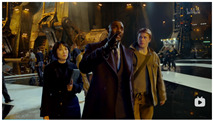	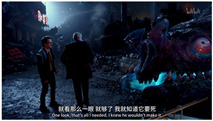	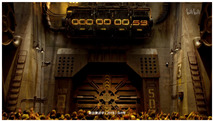	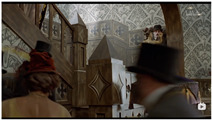	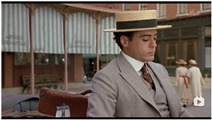	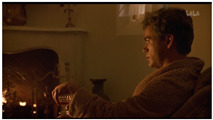
	Harry Potter and the Philosopher’s Stone			Catch Me If You Can		
Playback Time	40:50–42:34	77:41–78:32	147:23–148:18	40:46–41:59	61:23-62:16	126:09-126:59
Intensity Value	0.10,0.64,0.0,0.10, 0.11,0.05,0.0	0.07,0.39,0.0,0.05, 0.04,0.46,0.0	0.28,0.35,0.0,0.07, 0.0,0.30,0.0	0.0,0.61,0.0,0.07, 0.07,0.25,0.0	0.30,0.36,0.0,0.0, 0.0,0.34,0.0	0.29,0.44,0.0,0.0, 0.11,0.15,0.0
Intensity Figure	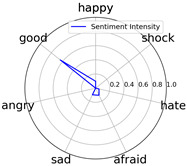	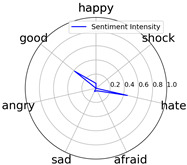	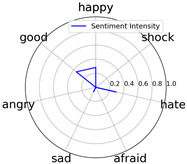	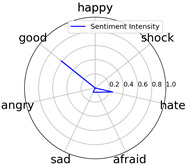	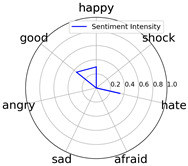	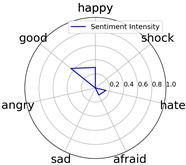
Film Plot	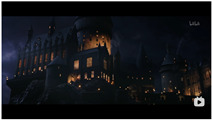	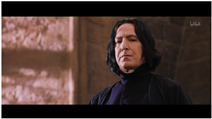	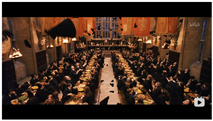	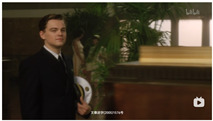	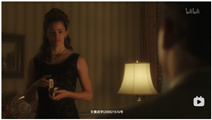	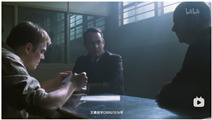
	Blood Diamond			Secret Superstar		
Playback Time	60:23–61:30	72:10–73:16	108:04–108:57	96:00–97:10	110:23–111:33	134:21–135:18
Intensity Value	0.12,0.46,0.0,0.06, 0.04,0.32,0.0	0.15,0.51,0.0,0.0, 0.09,0.19,0.06	0.09,0.35,0.0,0.06, 0.0,0.49,0.0	0.20,0.34,0.0,0.14, 0.04,0.20,0.07	0.03,0.25,0.0,0.16, 0.04,0.53,0.0	0.26,0.42,0.0,0.03, 0.06,0.21,0.02
Intensity Figure	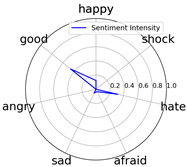	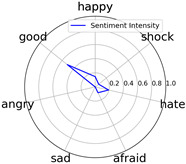	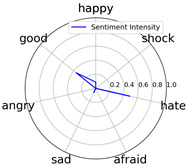	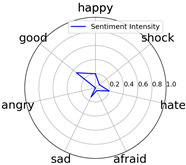	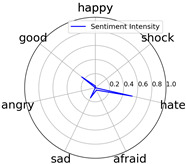	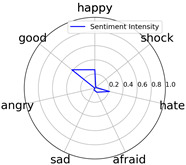
Film Plot	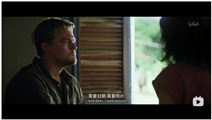	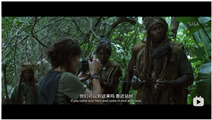	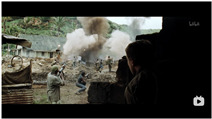	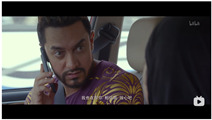	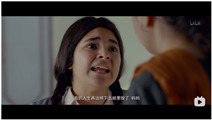	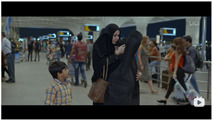

## Data Availability

The dataset and parameter configuration used to support the findings of this study are included within the article.
